# Indian Spices for Healthy Heart - An Overview

**DOI:** 10.2174/157340310793566172

**Published:** 2010-11

**Authors:** Hannah R Vasanthi, R.P Parameswari

**Affiliations:** Herbal & Indian Medicine Research Laboratory, Dept. of Biochemistry, Sri Ramachandra University, Chennai, India

**Keywords:** Spices, anti-inflammatory, anti-proliferative, anti-hypercholesterolemia, cardiovascular disease, diabetes.

## Abstract

Spices were some of the most valuable items of trade in the ancient and medieval world. Herbalist and folk practitioners have used plant remedies for centuries, but only recently have scientist begun to study the powers of common herbs and spices. In the current set-up, the anti-proliferative, anti-hypercholesterolemic, anti-diabetic, anti-inflammatory effects of spices have overriding importance, as the key health concern of mankind nowadays is diabetes, cardio-vascular diseases, arthritis and cancer. Spices or their active compounds could be used as possible ameliorative or preventive agents for these health disorders. Spices are rich in antioxidants, and scientific studies suggest that they are also potent inhibitors of tissue damage and inflammation caused by high levels of blood sugar and circulating lipids. Because spices have very low calorie content and are relatively inexpensive, they are reliable sources of antioxidants and other potential bioactive compounds in diet. This review outlines the role of some spices used in the Indian kitchen for its flavour and taste which are potential to maintain a healthy heart.

## INTRODUCTION

Currently there has been an augmented interest globally to identify compounds isolated from natural products that are pharmacologically effective and have low or no side effects for use in preventive medicine and the food industry. Alexander the Great's campaigns in Central Asia around 330 B.C. are often credited for the dissemination and adoption of herbs and spices among many cultures because they introduced Asian, Persian, Indian, and Greek cultures and ideas [1, 2]. Early records indicate that herbs and spices were used as medicines in ancient Egypt and Asia and as food preservatives in ancient Rome and Greece [3]. Herbs and spices continued to be used during the middle ages for flavoring, food preservation, and/or medicinal purposes [4]. In countries like India where poverty and malnutrition is unbridled, knowledge of plant derived antioxidants and spices could reduce the cost of health care. India has a rich history of using various herbs, spices and herbal components for treating various diseases [5]. It has been believed for some time that dietary factors play a key role in the development of some human diseases, including cardiovascular disease. Several herbs and spices of culinary origin were included in the “approved” monographs, such as caraway oil and seed, cardamom seed, cinnamon bark, cloves, coriander seed, dill seed, fennel oil and seed, garlic, ginger root, licorice root, mint oil, onion, paprika, parsley herb and root, peppermint leaf and oil, rosemary, sage, thyme, turmeric root, and white mustard seed [6]. The use of herbal medicine has skyrocketed over the last 10 years, with out-of-pocket costs estimated at more than $5 billion in the United States alone. The following review of herbal medicines affecting the cardiovascular system is based on information gleaned from the scientific literature. Most herbal medicinals have multiple effects modulating the cardiovascular system [7]. In the traditional Indian system of medicine *Ayurveda* and* Siddha *various spices and herbs are described to possess medicinal properties, such as being antithrombotic, antiatherosclerotic, hypolipidemic, hypoglycemic, anti-inflammatory, antiarthritic, etc [8].

Spices were some of the most valuable items of trade in the ancient and medieval world. Herbalist and folk practitioners have used plant remedies for centuries, but only recently have scientist begun to study the powers of common herbs and spices. Spices are rich in antioxidants, and a scientific study suggests they are also potent inhibitors of tissue damage and inflammation caused by high levels of blood sugar and circulating lipids. Due to their phenol content these are able to block the formation of compounds that contribute to damage caused by metabolic disorders. Because spices have very low calorie content and are relatively inexpensive, they are reliable sources of antioxidants and other potential bioactive compounds in diet [9]. The major focus of this review is on the role that spices play an inevitable role in the management of heart diseases, serving as agents both for prevention and treatment. Overall the review suggests "adding spice to our life" may serve as a delicious and sensible way to maintain a healthy heart.

## GARLIC

Several epidemiologic studies have indicated that certain diets are associated with low risk of cardiovascular disease and that these diets are rich in fruits, herbs and spices; the common spice among them is garlic [10].Garlic (*Allium sativum*) is believed to have originated in Central Asia and belongs to the Alliacae family. It is used universally as a flavoring agent, traditional medicine, and a functional food to enhance physical and mental health. Over the centuries, garlic has acquired a unique position in the myths of many cultures as an appalling prophylactic and therapeutic medicinal agent. It has been quoted in the *Egyptian Codex Ebers*, a 35-century-old document, as useful in the treatment of heart disorders, tumors, worms, bites and other ailments. Hippocrates and Pliny the Elder were both promoters of the intrinsic worth of garlic [11]. The first-century Indian physician Charaka (3000 BC), the father of Ayurvedic medicine, claimed that garlic acts as a heart tonic by maintaining the fluidity of blood and strengthens the heart [12]. Over the last 20 years, this important and exciting role of garlic has been and continues to be confirmed by basic and clinical research reports from around the world.

Garlic has been advocated for the prevention of heart disease [13]. Epidemiologic studies show an inverse correlation between garlic consumption and progression of cardiovascular disease. Cardiovascular disease is associated with multiple factors such as raised serum total cholesterol, raised LDL and an increase in LDL oxidation, increased platelet aggregation, hypertension, and smoking [14]. Garlic has been shown to inhibit enzymes involved in lipid synthesis, decrease platelet aggregation, prevent lipid peroxidation of oxidized erythrocytes and LDL, increase antioxidant status, and inhibit angiotension-converting enzyme [11]. Allicin, an active compound of garlic showed significantly lowered formation of fatty streaks in the aortic sinus [15]. Kleijnen *et al.* [16] showed that it also enhance blood fibrinolytic activity. Another *in-vitro* study shows that water – soluble organosulphur compound S –allyl cysteine present in aged garlic extract is a potent inhibitor of cholesterol synthesis [17, 18]. These findings have also been addressed in clinical trials too. The studies point to the fact that garlic reduces cholesterol, inhibits platelet aggregation, reduces blood pressure, and increases antioxidant status [14]. There is level III-3 evidence (National Health and Medical Research Council [NHMRC] levels of evidence) that consuming a half to one clove of garlic (or equivalent) daily may have a cholesterol-lowering effect of up to 9%. There is level III-1 evidence that 7.2 gm of aged garlic extract has been associated with anticlotting (in-vivo studies), as well as modest reductions in blood pressure (an approximate 5.5% decrease in systolic blood pressure) [19]. Warshafsky *et al.* [20] showed that an average of 900 mg garlic/ day could decrease total serum cholesterol levels by approximately 9%. Its consumption has been shown to have antiatherosclerotic activity to increase high-density lipoprotein (HDL) levels, which may help to remove excess cholesterol from arterial tissue in animal models and human cell cultures. It has been reported to have lipid-and blood pressure-lowering action, as well as antiplatelet, antioxidant, and fibrinolytic effects [21]. Garlic educes nitric-oxide-dependent relaxation in pulmonary arteries [22, 23]. Recently, Subhendhu *et al.* [24] have observed that freshly crushed garlic exerts superior cardioprotective activity than processed garlic. Their results show that freshly crushed garlic fed rats displayed significantly greater phosphorylation of antiapoptotic ERK1/2 proteins, reduced Bax/Bcl-2 ratio, and reduced phosphorylation of proapoptotic p-38MAPK and JNK. It enhanced redox signaling by increasing p65 subunit of NFκB, Nrf2, and enhanced GLUT 4, PPARα, and PPARδ. Also the survival signaling network consisting of Akt-FoxO1 was increased in the freshly crushed garlic treated hearts. Thus, to conclude it could be stated that garlic has multiple properties in the prevention of cardiovascular diseases and has to be taken as dietary supplement for prevention of CVD.

## CURCUMIN

Curcumin is an inevitable spice as well medicine used in the ancient traditional medicinal systems like Siddha and Ayurveda whose history goes back to over 5000 years. In Indian and Chinese medicines, turmeric was used as an anti-inflammatory agent to treat gas, colic, toothaches, chest pains, and menstrual difficulties. This spice was also used to help with stomach and liver problems, to heal wounds and lighten scars, and as a cosmetic [25]. In a natural mutant model of obesity, turmeric (at 1 and 5% of the diet) had significantly reduced cholesterol and triglyceride concentrations while increasing HDL cholesterol, within 4 weeks. Further evidence indicates that it reduces the oxidation of LDL, blood glucose and renal lesions in diabetes. In addition, it has been demonstrated to reduce platelet aggregation, cyclooxegenase, thromboxane, smooth muscle cell proliferation and endothelial dysfunction. Both turmeric and curcumin, due to their antioxidant and anti-inflammatory activity, have been demonstrated to counteract several disorders such as myocardial infarctions, chronic inflammatory lung diseases, pancreatitis, inflammatory bowel diseases, neurodegenerative diseases, hepatic and lung damages as well as muscle injuries and cystic fibrosis [26] Curcumin can also impact on the process of cataractogenesis and delays galactose-induced cataracts formation in rats [27].

Studies show that the preventive mechanisms of curcumin regarding with heart disease is multi targeted. The antioxidant effects of curcumin have been shown to attenuate adriamycin-induced cardiotoxicity [28] and may prevent diabetic cardiovascular complications [29] antithrombotic, [30] anti-proliferative, [31] and anti-inflammatory effects of curcumin and the effect of curcumin in decreasing the serum cholesterol level may protect against the pathological changes seen in atherosclerosis [32]. Curcumin, present in turmeric inhibits NF-κB transcriptional factor, through inhibition of IKK, a kinase which is needed for NF-kB activation; improve the surge of pro-inflammatory cytokines during cardiopulmonary bypass (CBP) and decreases cardiomyocytic apoptosis after global cardiac ischemia/reperfusion injury [33]. More extensive research regarding the effect of curcumin on the cardiovascular diseases in both humans as well as in animals is defensible.

## GINGER

Ginger (*Zingiber officinale* Roscoe, Zingiberacae) is a medicinal plant that has been widely used in Chinese, Ayurvedic and Tibb-Unani herbal medicines all over the world, since ancient times, for a wide range of unrelated ailments that include arthritis, rheumatism, sprains, muscular aches, pains, sore throats, cramps, constipation, indigestion, vomiting, hypertension, dementia, fever, infectious diseases and helminthiasis [34]. The health benefits of ginger documented over 2,000 years ago indicates that ginger has various medicinal properties. The active ingredient in ginger is gingerol, a compound that's thought to relax blood vessels, stimulate blood flow and relieve pain. Other potentially active compounds present in ginger are phenolic compounds – shogaols and gingerols; sesquiterpenes - bisapolene, zingiberene, zingiberol, sesquiphellandrene, curcurmene and other compounds are 6-dehydrogingerdione, galanolactone, gingesulfonic acid, zingerone, geraniol, neral, monoacyldigalactosylglycerols, gingerglycolipids [35].

Ginger is also an anti-inflammatory agent, which means it may be useful in fighting heart disease, cancer, Alzheimer's disease and arthritis. Antimicrobial, anti thrombotic, anti-inflammatory and anticancer activity have also been reported [36].

In a placebo controlled clinical trial patients administered with single dose of 10 g powdered ginger administered to Coronary artery diseased patients produced a significant reduction in platelet aggregation induced by the two agonists, but did not affect the blood lipids and blood sugar [37]. Hyperlipidemic rabbits when challenged with a 50% ethanolic extract of ginger showed a reduction in total cholesterol and serum LDL-cholesterol. A reduction in HDL ratio was seen in atherofed rabbits compared with controls which were restored when challenged with the *Zingiber* extract. An atherogenic index of 4.7 was brought down to 1.2 using plant products. The tissue lipid profiles of liver, heart and aorta showed similar changes to those noticed in serum lipids [38]. *Zingiber* extract feeding in diabetic rats increased the fecal excretion of cholesterol thus suggesting a modulation of absorption. Ethanolic extract of ginger can protect the tissues from lipid peroxidation. The extract also exhibit significant lipid lowering activity in diabetic rats [39].

Consumption of ginger extract inhibited the progression of aortic atherosclerosis in atherosclerotic, apolipoprotein- E deficient mice. This effect was associated with a significant reduction in the plasma and LDL cholesterol levels, with a parallel reduction in the oxidative response of macrophages, and reduced LDL atherogenic modifications (oxidation and aggregation). The anti-atherogenicity of ginger extract could also be attributed to its direct antioxidative effects on macrophages as well as on plasma LDL [40]. Guh *et al.* [41] showed that Gingerol isolated from Zingiber inhibit platelet function by inhibiting thromboxane formation. Feeding rats with ginger significantly elevated the activity of hepatic cholesterol-7α-hydroxylase, the rate-limiting enzyme in bile acids biosynthesis, thereby stimulating cholesterol conversion to bile acids, resulting in elimination of cholesterol from the body [42]. In addition, a pure constituent from ginger [E-8 beta, 17 epoxylabd-12-ene-15, 16-dial (ZT)], was shown to inhibit cholesterol biosynthesis in homogenated rat liver [43]. In 20 healthy young male volunteers, ginger supplementation (5gms daily) significantly inhibited the platelet aggregation induced by ADP (adenosine diphosphate) and epinephrine [44]. In human volunteers who took a huge (10 gram) one-time dose of dried ginger, there was a marked inhibition of platelet aggregability [37]. Another study showed no significant impact of fresh or cooked ginger (doses up to 15 grams of fresh ginger or 40 grams of cooked ginger) on thrombotic activity or platelet thromboxane production [45]. The above mentioned reports evidence the cardioprotective potential of ginger and thus could be used for the prevention and treatment of cardiovascular disorders by adding them in our day to day dietary intake.

## BLACK PEPPER

Pepper is an extensively used spice both in Eastern and Western food. It has an impressive antioxidant and antibacterial effect and helps with digestion and weight loss because it stimulates the breakdown of fat cells. Black pepper is considered as the king of spices, as it fetches the highest return as judged from the volume of international trade. Black pepper or its active principle piperine has been experimentally demonstrated by a number of independent investigators to possess diverse physiological effects. Piperine has been demonstrated in *in vitro *studies to protect against oxidative damage by inhibiting or quenching free radicals and reactive oxygen species. Black pepper or piperine treatment has also been evidenced to lower lipid peroxidation *in vivo* and beneficially influence cellular thiol status, antioxidant molecules and antioxidant enzymes in a number of experimental situations of oxidative stress [46]. Wakade *et al.* showed that the methanolic extract of *Piper longum* exhibits a significant protection against adriamycin induced cardiotoxicity by virtue of its antioxidant and free radical scavenging capacity. Black pepper has been reported to influence lipid metabolism predominantly by mobilization of fatty acids [47]. An *in vivo* study on hypolipidemic effect of black pepper (*Piper nigrum* Linn.) in high fat diet fed rats treated with black pepper as well as piperine showed remarked decrease in the levels of cholesterol (both the free and ester cholesterol fractions), free fatty acids, phospholipids and triglycerides. Moreover, supplementation of the high fat fed rats with black pepper elevated the concentration of high density lipoprotein-cholesterol (HDL-c) and reduced the concentrations of low density lipoprotein-cholesterol (LDL-c) and very low density lipoprotein-cholesterol (VLDL-c) in the plasma as compared with the levels in unsupplemented high fat fed rats [48]. Vanadium compounds also promote cardiac function by activating Akt signaling through inhibition of protein tyrosine phosphatases. Black pepper, being rich in containing vanadium in it thus elicits cardiac functional recovery in myocardial infarction and pressure overload–induced hypertrophy [49].

## CINNAMON

*Cinnamon* belongs to genus *Cinnamomum*, family *Lauraceae *which is distributed in India, Egypt, China, Srilanka and Australia. *Cinnamon *leaves and bark are used extensively as spices in food or to produce essential oils [50]. Studies have shown the antioxidant and antimicrobial potential [51], the antidiarrhoeal activity of Cinnamon is also well documented. The ‘Indian Materia Medica’ [52] and the ‘Indian Medicinal Plants – A Compendium of 500 species’ [53] classifies cinnamon as a herbal drug which has cardiovascular effects. Wang *et al.* [54] in their study using guinea pig heart showed that a decoction of cinnamon increased coronary blood flow and provoked pituitrin induced reduction of blood flow. Also it reduced peripheral vascular resistance, suggesting an undeviating vasodilation of peripheral vessels. Increased cardiac contractile force and beating rate was also exerted by cinnamaldehyde, which is present in cinnamon. Circulatory stimulant effects of cinnamon have been reported in several books on medicinal plants and also in *Ayurveda* [55, 56]. Dietary cinnamon increases bilary secretion of cholesterol and phospholipids without affecting the bile content (Sambaiah and Sreenivasan [42]. Sharma *et al.* [57] studied the effect of a 50% alcoholic extract of cinnamon on rats and reported a significant anti-hypercholesterolemic action and reduced serum triglyceride level at a single dose of 250 mg/kg body weight. Suppression of total serum cholesterol, triglycerides, phospholipids and low density lipoprotein levels was observed in another investigation using triton WR-1339-induced hyperlipidaemic rats [58]. The same group in their extension study with 80% methanolic extract and its chloroform fraction of different species of *Cinnamomum *observed that the extracts suppressed the elevated serum total cholesterol and triglyceride levels in corn oil-induced hyperlipidaemic rats. The chloroform fraction exhibited remarkable inhibitory effects on HMG-CoA reductase, an enzyme that catalyses cholesterol biosynthesis. Kamal [59] *et al.* showed in their study that cinnamon extract improved lipid profile by extensively decreasing total cholesterol, triglycerides and LDL – C levels with increasing serum HDL – C. It also hampers HMG-CoA reductase activity in liver thereby lowering the Cholesterol levels. Cinnamon activates PPARγ resulting in improved insulin resistance and reduced fasted LDL-c, thereby managing obesity related hyperlipidemia and also increases NO levels, which is a potent Vasodilator. Cinnamon demonstrated significant ability to inhibit initiation as well as propagation of lipid peroxidation due to their polyphenol content, strong reducing power and superoxide radical scavenging activity [60]. Cinnamon exhibited linear dose-dependent Nitric oxide suppressing effect without any effect upon cell viability [8]. Peroxisome proliferator-activated receptors (PPARs *γ* and *α*), one among the transcriptional factors involved in the regulation of insulin resistance and adipogenesis is found to get activated, resulting in improved insulin resistance, reduced fasted glucose, FFA, LDL-c, and AST levels in high-caloric diet-induced obesity (DIO) and *db/db *mice fed with cinnamon water extract [61].

## CORIANDER

Coriandrum sativum (Coriander) has been documented as a traditional treatment for cholesterol and diabetes patients. It has a long history as a traditional medicine [62]. The seeds of coriander have a remarkable hypolipidemic action. The levels of total cholesterol and triglycerides decreased significantly in the tissues of the animals of the experimental group which received coriander seeds. Significant increases in -hydroxy, -methyl glutaryl CoA reductase and plasma lecithin cholesterol acyl transferase activity were noted in the experimental group. The level of LDL + VLDL cholesterol decreased while that of HDL cholesterol increased in the experimental group compared to the control group. The increased activity of plasma LCAT enhanced hepatic bile acid synthesis and the increased degradation of cholesterol to fecal bile acids and neutral sterols appeared to account for its hypocholesterolemic effect [63]. Thrombosis, an important event in cardiovascular diseases, can be fatal if platelet aggregation takes place in the narrowed lumen of arteries, causing an impairment of blood flow to the heart. Attempts have been made to study the antiplatelet activity of leaf spice extracts, as these are rich sources of natural antioxidants. Aqueous extracts of coriander leaf and curry leaf were tested on human platelets over a wide range of concentrations. Both these leaf spice extracts inhibited human platelet aggregation [64]. Another *in vivo* study shows that there is a remarkable decrease in the levels of TC, TG, TAG and LDL-c in plasma, also there was a significant increase in the levels of HDL-c observed in the cholesterol-rich (1%) basal diet fed rats treated with coriander seed oil [65].

## CONCLUSION

To summarize, spices are heterogeneous collections of a wide variety of volatile and non-volatile staple dietary additives. India with its wide climatic conditions and topographical features naturally possesses wide variety of spices which are being used in the diet. The above discussed spices namely garlic, pepper, coriander, ginger, turmeric, cinnamon are commonly used spices in Indian delicacies. These spices turn an ordinary meal to an extraordinary experience. They have a diverse array of natural phytochemicals that have complementary and overlapping actions. As several metabolic diseases and age-related degenerative disorders such as cardiovascular disorders are closely associated with oxidative processes in the body, the use of herbs and spices as a source of antioxidants to combat oxidation warrants further attention. From a dietary perspective, the functionality of herbs and spices will be exposed through consideration of their properties as foods. As with most foods, the real benefits of including them in the diet are likely to emerge with a better understanding of the attributes of health that are best supported by food, and in methodological developments addressing the evidence base for their effects. These developments are well underway through evidence-based frameworks for substantiating health claims related to foods for a healthy heart. At present, recommendations are warranted to support the consumption of foods rich in bioactive components, such spices. With time, we can expect to see a greater body of scientific evidence supporting the benefits of spices in the overall maintenance of a healthy heart which is the most important organ for every beat of life and protection from diseases of the heart.

## Figures and Tables

**Fig. (1):**
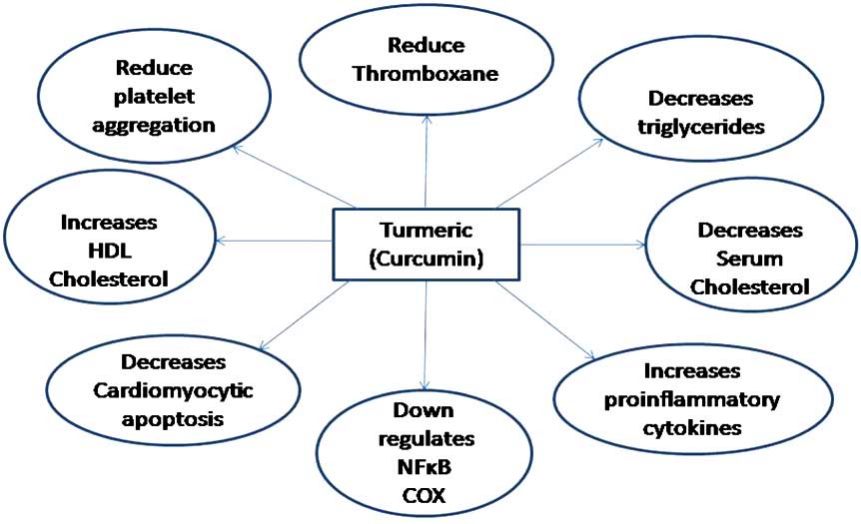
Potential effects of Turmeric (Curcumin) on the prevention of cardiovascular diseases.
